# Origin and Diet of the Prehistoric Hunter-Gatherers on the Mediterranean Island of Favignana (Ègadi Islands, Sicily)

**DOI:** 10.1371/journal.pone.0049802

**Published:** 2012-11-28

**Authors:** Marcello A. Mannino, Giulio Catalano, Sahra Talamo, Giovanni Mannino, Rosaria Di Salvo, Vittoria Schimmenti, Carles Lalueza-Fox, Andrea Messina, Daria Petruso, David Caramelli, Michael P. Richards, Luca Sineo

**Affiliations:** 1 Department of Human Evolution, Max Planck Institute for Evolutionary Anthropology, Leipzig, Germany; 2 Institute of Evolutionary Biology, Consejo Superior de Investigaciones Científicas, Universitat Pompeu Fabra, Barcelona, Spain; 3 Dipartimento di Biologia Evoluzionistica, Laboratori di Antropologia, Università degli Studi di Firenze, Florence, Italy; 4 Museo Archeologico Regionale “Antonino Salinas”, Palermo, Italy; 5 Dipartimento di Biologia Ambientale e Biodiversità, Università degli Studi di Palermo, Palermo, Italy; 6 Dipartimento di Scienze della Terra e del Mare, Università degli Studi di Palermo, Palermo, Italy; 7 Department of Anthropology, University of British Columbia, Vancouver, Canada; Museo Nazionale Preistorico Etnografico ‘L. Pigorini’, Italy

## Abstract

Hunter-gatherers living in Europe during the transition from the late Pleistocene to the Holocene intensified food acquisition by broadening the range of resources exploited to include marine taxa. However, little is known on the nature of this dietary change in the Mediterranean Basin. A key area to investigate this issue is the archipelago of the Ègadi Islands, most of which were connected to Sicily until the early Holocene. The site of Grotta d’Oriente, on the present-day island of Favignana, was occupied by hunter-gatherers when Postglacial environmental changes were taking place (14,000-7,500 cal BP). Here we present the results of AMS radiocarbon dating, palaeogenetic and isotopic analyses undertaken on skeletal remains of the humans buried at Grotta d’Oriente. Analyses of the mitochondrial hypervariable first region of individual Oriente B, which belongs to the HV-1 haplogroup, suggest for the first time on genetic grounds that humans living in Sicily during the early Holocene could have originated from groups that migrated from the Italian Peninsula around the Last Glacial Maximum. Carbon and nitrogen isotope analyses show that the Upper Palaeolithic and Mesolithic hunter-gatherers of Favignana consumed almost exclusively protein from terrestrial game and that there was only a slight increase in marine food consumption from the late Pleistocene to the early Holocene. This dietary change was similar in scale to that at sites on mainland Sicily and in the rest of the Mediterranean, suggesting that the hunter-gatherers of Grotta d’Oriente did not modify their subsistence strategies specifically to adapt to the progressive isolation of Favignana. The limited development of technologies for intensively exploiting marine resources was probably a consequence both of Mediterranean oligotrophy and of the small effective population size of these increasingly isolated human groups, which made innovation less likely and prevented transmission of fitness-enhancing adaptations.

## Introduction

Hunter-gatherer subsistence strategies in coastal Mediterranean environments during the closing stages of the Pleistocene and in the early Holocene appear to have been based overwhelmingly on the exploitation of terrestrial animals with minor contributions by coastal resources, such as marine molluscs [1]–[2]. In some areas of the Mediterranean Basin, however, faunal assemblages from the Upper Palaeolithic include remains of fish and marine mammals [3]. Zooarchaeological studies on Mesolithic assemblages suggest that the contribution of marine resources to human subsistence increased in the early Holocene [2], [4].

Data on the diets of coastal hunter-gatherers have also been obtained from carbon and nitrogen stable isotope analyses on collagen from skeletal remains, which for most of the Mediterranean humans analysed (Table 1) suggest little or no consumption of marine foods [5]–[9], thereby confirming the dietary reconstructions based on the faunal remains. In a few cases higher levels of marine food consumption have been recorded through isotope analyses [7], [10]–[11], albeit not reaching the levels of contemporary individuals from the European coasts of the Atlantic Ocean [12]–[13].

**Table 1:** Carbon and nitrogen isotope values from bone collagen of Mediterranean late Upper Palaeolithic (17–11 ka cal BP) and Mesolithic (10-8 ka cal BP) humans.

Period	Site	Individuals	δ^13^C (‰)	δ^15^N (‰)	Source
Upp. Pal.	Grotta d’Oriente (Sicily)	Oriente C	−19.3	11.0	[14]
Upp. Pal.	Grotta Addaura Caprara (Sicily)	Addaura 1	−19.7	9.6	[8], [9]
Upp. Pal.	Grotta di San Teodoro (Sicily)	3	−19.7±0.4	12.0±0.4	[8]
Upp. Pal.	Grotta del Romito (Italy)	8	−19.5±0.3	10.1±1.1	[14]
Upp. Pal.	Arene Candide (Italy)	2	−19.5±0.8	9.0±0.1	[5]
Mesolithic	Grotta d’Oriente (Sicily)	2	−18.4±0.8	11.0±0.5	this study
Mesolithic	Grotta Addaura Caprara (Sicily)	2	−19.5±0.2	9.2±0.7	[9]
Mesolithic	Grotta Molara (Sicily)	2	−19.9±0.5	8.8±2.3	[9]
Mesolithic	Grotta dell’Uzzo (Sicily)	2	−21.0±0.0^*^	10.6±0.2^*^	[5]
Mesolithic	El Collado (Spain)	9	−18.3±0.7	10.3±1.2	[10]
Mesolithic	Vela Spilja – Vela Luka (Croatia)	4	−18.6±0.6	9.2±1.0	[7]
Mesolithic	Pupićina Cave (Croatia)	2	−19.2±0.1	10.5±0.3	[7]
Mesolithic	Pupićina Cave (Croatia)	4	−19.3^**^	10.6^**^	[6]
Mesolithic	Monte Leone (Corsica)	1	−18.0	9.9	[11]
Mesolithic	Araguina Sennola (Corsica)	1	−18.8	10.6	[11]

(*)the C/N ratios of the human bone collagen from Grotta dell’Uzzo are at the limit for well-preserved collagen ( = 3.6). In the absence of other published data on the quality of the collagen (%C, %N, %yield) analysed by Francalacci [5], the isotope ratios of these individuals should be considered with caution. The low δ^13^C values might, in fact, be due to contamination.

(**)the standard deviation could not be calculated because the raw data was not published by Paine et al. [6].

The variability in the reliance on marine resources by Mediterranean and Atlantic hunter-gatherers has been explained by different authors as the consequence of the low primary productivity of the Mediterranean Sea [7]–[10], [14]. The limited tidal range of this land-locked sea is another reason for the lower biomass available along its shores [15]. Excluding the westernmost part of the Mediterranean Sea, which is known as the Alboran Sea, and which is characterized by higher primary productivity than the rest of the Basin, the highest productivity occurs in coastal areas with wide continental shelves, such as the Gulf of Lyon, the north Adriatic Sea and the Gulf of Gabes [16]. These areas are characterized by gently-shelving soft-bottom shores, lagoons and brackish water environments, which are generally more productive than rocky shores. Bailey and Flemming [17] argue that areas such as these are more likely to have been flooded by Postglacial sea level rise. Reconstructions of the role of marine resources in the diet of hunter-gatherers living on these more productive coasts might be biased, because sites along them might have been submerged by rising sea levels, particularly those which might contain higher proportions of marine food refuse.

**Figure 1:**
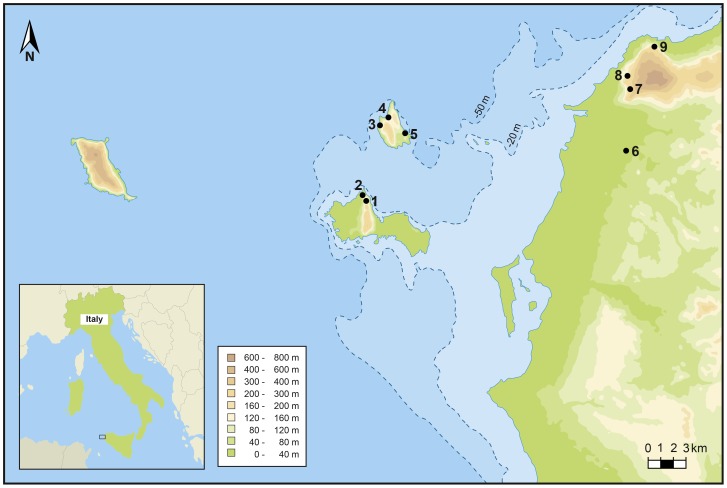
Location of Upper Palaeolithic and Mesolithic sites on the Ègadi Islands and in NW Sicily. These cave sites include: Grotta d’Oriente (1) and Grotta dell’Ucceria (2) on the island of Favignana; Grotta di Punta Capperi (3), Grotta di Cala dei Genovesi (3), Grotta Schiacciata (4) and Grotta di Cala Calcara (5) on the island of Levanzo; Grotta Maiorana (6), Riparo San Francesco (7), Grotta Martogna (8), Grotta Emiliana (9) and Grotta Maltese (9) on the mainland of Sicily. No late Pleistocene or early Holocene site has been discovered on the westernmost island of the archipelago, Marettimo, and on the two islets of Maraone and Formica, between Levanzo and the mainland. Taking into account that the coast of NW Sicily has been tectonically stable during the period in question and using the sea level curve published by Lambeck et al. [20], it can be hypothesized that Levanzo became an island sometime between 9,000 and 8,500 cal. BP (as modelled by Mannino et al. [74]), while Favignana was fully isolated around 7,000 calibrated years BP and was a peninsula for part of the early Holocene. The later Mesolithic hunter-gatherer (Oriente X), whose remains have been recovered at Grotta d’Oriente, lived at a time (ca. 9,500 cal. BP) when the sea level was around −41 m. At this time the shoreline was somewhere between the −50 m and −20 m bathymetry contour lines and Levanzo was probably a small peninsula.

One way of dealing with this bias in the archaeological record is to undertake isotope analyses on skeletal remains of humans that lived in areas affected by dramatic sea level rise. Carbon and nitrogen isotope analyses are effective at establishing the contribution of marine versus terrestrial protein in human diets [18], as testified by the above-mentioned studies. An ideal area to adopt this approach is the present-day archipelago of the Ègadi Islands, off western Sicily (Fig. 1). Two of these islands (Favignana and Levanzo) were connected to mainland Sicily until the first few millennia of the Holocene [19]–[20]. As reviewed by Tusa [21], cave sites containing Upper Palaeolithic and Mesolithic lithic and faunal assemblages, are present on Levanzo and Favignana. Of these sites, Grotta d’Oriente, on the island of Favignana, was the only cave where prehistoric burials attributable to the late Upper Palaeolithic [22] and Mesolithic [23] were found. Here we present the results of biomolecular analyses (palaeogenetic and isotopic) on human remains from the Mesolithic burials of Grotta d’Oriente, which throw new light on the origin of the hunter-gatherers in question and on how they responded to changing environmental conditions in Mediterranean coastal habitats during periods of dramatic sea level rise, such as those that in the Postglacial led to the progressive isolation of Levanzo and Favignana [19]–[20].

### Grotta d’Oriente

#### The site and its setting

The major substrata of the Ègadi Islands, including Favignana, are composed of carbonatic and clastic-terrigenous deposits Mesozoic-Tertiary in age overlain in discordance by Plio-Pleistocene terrains [24]. Around the Late Glacial Maximum and up to the early Holocene, sea levels were low enough to expose the fossiliferous calcarenite of Early Pleistocene age connecting all the Ègadi Islands with the possible exception of Marettimo to the western Sicilian coast. Under these favourable conditions, terrestrial mammals and humans reached Levanzo and Favignana, as documented by the bone assemblages recovered from cave deposits on these islands [25]–[28].

**Figure 2:**
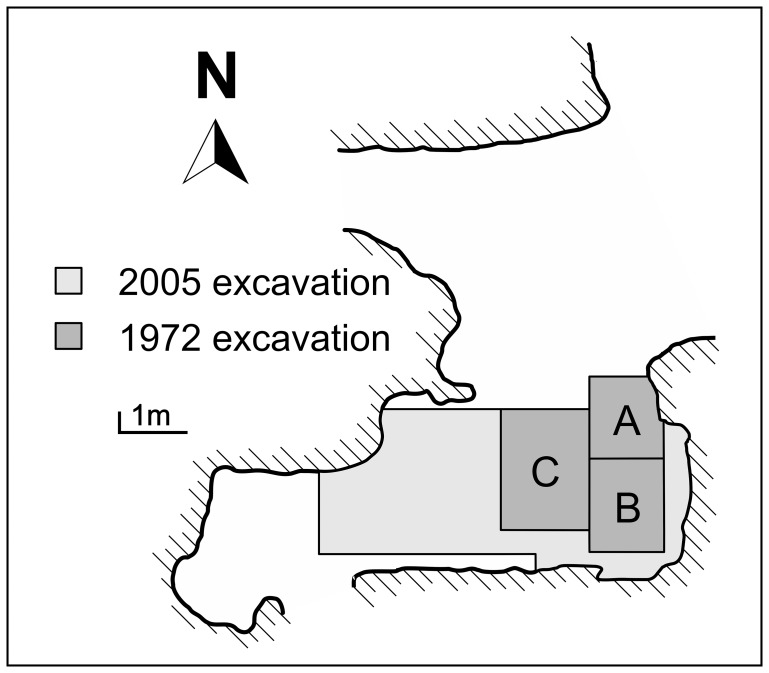
Plan of Grotta d’Oriente illustrating areas and trenches excavated during the 1972 and 2005 campaigns.

Numerous caves are present on Favignana around Montagna Grande [29], many of which contain traces of human occupation dating to the Late Glacial (Upper Palaeolithic) and early Holocene (Mesolithic). Grotta d’Oriente, on the north-eastern slopes of Montagna Grande at 50 m a.s.l., is a cave with a small external chamber and a large internal cavity (Fig. 2). In 1972 Giovanni Mannino excavated deposits in the outer chamber, over an area of 6 m^2^ in three trenches called A, B and C, establishing the presence of a sequence spanning from the late Upper Pleistocene to the late Holocene [23]. The anthropogenic sequence explored by Mannino stopped at around 1.40 m of depth, below which only a few bone fragments of Pleistocene fauna, not associated to human activities, were recovered. The deposits above 1.40 m contained artefacts and fauna from the Mesolithic (defined ‘Epi-Palaeolithic’ by the excavator), Neolithic, Copper Age, Bronze Age and later periods. A burial, which we will refer to hereafter as Oriente A, dug into the late Pleistocene deposits was intercepted at around 1.14 m of depth in Trench B, while in Trench C, at a depth of 1.10 m, a second burial (Oriente B) was uncovered. On stratigraphic grounds, Oriente A could be late Upper Palaeolithic or early Mesolithic, while Oriente B should be Mesolithic [23]. The deposits excavated in 1972 mainly date back to the Mesolithic (Trench B: 60–114 cm; Trench C: 40–180 cm) and, as reported by Mannino and Thomas [26], contained scarce remains of terrestrial mammals, such as red deer (*Cervus elaphus*), aurochs (*Bos primigenius*), wild boar (*Sus scrofa*) and European ass (*Equus hydruntinus*). Intertidal rocky shore molluscs of the genera *Patella* and *Osilinus* were the most frequently exploited marine resources during the Mesolithic, with limited evidence of fishing [26]. In Trench B (40–60 cm) and Trench C (14–40 cm) the excavations also explored levels which include domestic (i.e. *Ovis vel Capra*) and wild mammals (i.e. *C. elaphus*), as well as birds, fish (mainly dusky grouper, *Epinephelus marginatus*), reptiles, crustaceans, marine and terrestrial molluscs. On the basis of strong similarities with contemporary assemblages from sites such as Grotta dell’Uzzo [4], Grotta di Cala dei Genovesi [25] and Grotta Schiacciata [27], these deposits can be attributed to a period between the late Mesolithic and the early Neolithic.

To confirm the chronological attributions based on the material culture and fauna from the 1972 excavation, two shells of the marine mollusc *Osilinus turbinatus* from Trench B were AMS radiocarbon dated. One of the selected shells was recovered at 100–114 cm, immediately above the burials, thereby providing a *terminus ante quem* for their age, while the other is from the stratigraphic unit 40–60 cm and thus enabling us to verify if the attribution of this part of the sequence to the Mesolithic-Neolithic transition is correct. The radiocarbon determinations are reported in Table 2. The calibrated age for the shell from stratigraphic unit 100–114 cm (8,740-8,390 cal BP) confirms that this part of the deposit dates to the early Holocene climatic phase of the Boreal and suggests that burial Oriente B is early Mesolithic. The determination on the shell from 40–60 cm (7,510-7,260 cal BP) attributes this part of the deposit to the beginning of the Atlantic climatic phase (Holocene Climatic Optimum), confirming that this level accumulated during the Mesolithic-Neolithic transition or, possibly, in the early Neolithic [30].

**Table 2:** AMS radiocarbon dates on shells of the marine intertidal gastropod *Osilinus turbinatus*.

AMS radiocarbon laboratory number	Context	^14^C Date (BP)	Calendar age cal. BC (2σ)	Calendar age cal. BP (2σ)
OxA-15562	B40/60	6955±36	5570 (p. 95.4%) 5310	7510 (p. 95.4%) 7260
OxA-14256	B100/114	8159±37	6790 (p. 95.4%) 6440	8740 (p. 95.4%) 8390

The dates were performed at the Oxford Radiocarbon Accelerator Unit on specimens from the stratigraphic units 40–60 cm and 100–114 cm of Trench B (Grotta d’Oriente). The dates were calibrated with Oxcal 4.1. [48] using the Marine09 calibration curve [49] and the marine reservoir correction value (ΔR = 71.00±50) for Sicily [82].

Recent excavations undertaken at Grotta d’Oriente in 2005, contiguously to the trenches excavated in the 1970s by Mannino, confirmed that the stratigraphy of the cave is characterized by layers dating to four main phases: late Upper Palaeolithic (14,640-13,760 cal BP), Mesolithic (9,890-9,460 cal BP), Mesolithic-Neolithic transition or early Neolithic (7,990-7,750 cal BP), and Bronze Age [28], [31]. The 2005 excavations were conducted in a more systematic fashion compared to those of 1972 and according to the excavators managed to intercept short-term episodes associated with intense exploitation of coastal resources [31]. The results of this more recent campaign have only been published in a preliminary form, but it appears that terrestrial resources provided the bulk of the food [28], as previously shown by Mannino and Thomas [26]. A wider array of marine resources, and in particular fish, might have been exploited than is suggested by the range of taxa recovered from the excavations of 1972 [26]. Oxygen isotope analyses on shell carbonates of the intertidal gastropod *Osilinus turbinatus* suggest that in the Mesolithic the collection of marine molluscs was a markedly seasonal activity, limited to the coldest months of the year [32]. Overall, as at other Upper Palaeolithic and Mesolithic sites in NW Sicily and in the Mediterranean (Table 1), marine molluscs probably represented marginal foods in the diet of the occupants of Grotta d’Oriente, albeit constituting a source of nutrients that could not be obtained from other resources [9]. Other possible sources of nourishment were terrestrial gastropods, such as *Eobania vermiculata*, although it is not yet clear whether these molluscs were actually consumed [31].

#### The burials

Three burials have been unearthed at Grotta d’Oriente; two during the above-mentioned 1972 campaign (Oriente A and Oriente B) [33] and a third, Oriente C, during excavations in 2005 [22] (Fig. 2). Oriente A was intercepted at 1.14 m of depth in Trench B and partly in Trench A. This individual was only represented by a few cranial fragments and an incomplete mandible, attributable to an adult male [33]. An intact shell of the marine mollusc *Patella ferruginea*, with traces of ochre, was found next to the maxilla, while 10 perforated and worked shells of marine gastropods (*Luria lurida*) and bivalves (*Ostrea* cfr. *edulis*) were recovered below the cranium, in the area which must have been occupied by the clavicles and sternum [23], [26], [34]. A second individual, Oriente B, was found at around 1.10 m of depth in Trench C. This individual was almost complete, with good preservation of the cranial and post-cranial portions of the skeleton, the study of which has determined that Oriente B is an adult female [33]. The skeletal elements belonging to Oriente B include: the cranium, the mandible, numerous ribs, most of the vertebrae and of the appendicular skeleton. It should be noted, however, that the sternum, the right tibia, a few carpal and tarsal bones were missing. During the excavation of Oriente B, 8 perforated marine shells (5 *Luria lurida*, 1 *Conus mediterraneus* and 2 *Spondylus gaederopus*) were recovered in the area just below the clavicles [34], a position which was taken to indicate their use in a necklace [23].

The excavations of 2005, in the outer chamber of Grotta d’Oriente, unearthed a third burial (Oriente C) that had been cut in half during the excavations of 1972, roughly at the height of the wrists, by the southern section of Trench B [22]. For this reason, Oriente C, an adult female, is only represented by the upper half of the skeleton, while the hands, hips and lower limbs of this individual were not present. Another feature of this burial is that a femur was placed on the thorax between the shoulders, although, it is not clear how long after the initial burial the femur was moved and to which individual this bone belonged. Oriente C is being studied by another group of researchers and it is yet undated. A radiocarbon date on a charcoal fragment from the layer immediately below the burial suggests that it must be later than 12,132±80 BP (14,210-13,770 cal BP) and according to the excavators it is attributable to the Upper Palaeolithic and associated to the Late Epigravettian culture [22].

Lo Vetro and Martini [22] have proposed a reconstruction for the chronological succession of the three burials, resulting from observations made during the 2005 excavation campaign. According to these researchers, Oriente A and C were extremely close, if not contiguous. On the basis of stratigraphical observations, however, they hypothesize that Oriente C might have been slightly more recent than Oriente A, because the surface in which the former was deposed appeared to seal the top of the latter. Lo Vetro and Martini [22] also suggested that Oriente B might have been roughly contemporary to the other two burials or later, possibly Mesolithic.

In the course of the 1972 excavations, at least 40 human remains were also recovered outside of the burial contexts and these were identified as human during post-excavation work at the Museo Archeologico Regionale “Antonino Salinas” in Palermo. The only remain found in Trench A is an upper molar from 80–114 cm of depth. Twenty-nine remains originated from Trench B including: 1 right metatarsus from 80–100 cm; 2 vertebral fragments, 1 distal left humerus, 1 distal left radius, 1 distal right ulna, 4 metacarpals, 3 fragments of phalanges of the carpus, 1 small fragment of distal femur, 1 cuneiform, 4 metatarsals, 1 fragment of a phalanx from the tarsus and 9 small undetermined human bone fragments from 100–114 cm. Ten remains were recovered from Trench C: 1 fragment of a sternum, 1 vertebral spine fragment, 2 carpals, 1 metacarpal, 3 fragments of phalanges of the carpus and 1 phalanx of the tarsus from 40–75 cm; 1 vertebral fragment from 75–110 cm.

The remains from Trenches A and B are not compatible with Oriente B on archaeological and anatomical grounds. Oriente B was recovered in primary deposition at a similar depth but in Trench C, it is unlikely that any of the bones recovered in Trenches A and B belonged to this individual. This can also be excluded on anatomical grounds, given that the distal right ulna fragment recovered in Trench B at 100–114 cm is too large to be from the incomplete right ulna of Oriente B, which only lacks the styloid process. On the other hand, it cannot be excluded that some of the remains from Trenches A and B belonged to Oriente A, given that this individual was found mainly in Trench B immediately below the bones recovered in the same trench from 100–114 cm of depth and is only represented by the cranium and mandible. Judging by the photographic evidence published by Lo Vetro and Martini [22], it can probably be excluded that the distal left humerus, distal left radius and distal right ulna from Trench B (100–114 cm) belonged to Oriente C, which was unearthed immediately to south of Trench B in the strip of the trench opened during the 2005 excavations, flanking the back wall of the cave (Fig. 2).

In summary, the distal left humerus and radius, and distal right ulna from Trench B clearly do not belong to Oriente B and C, but might be pertinent to Oriente A or even to a fourth individual. In the absence of conclusive evidence on the individual to which these three bones belonged we have attributed them to Oriente X. The other identifiable bones from Trench B might belong to Oriente A or even Oriente C, but this will not be ascertainable until the study of Oriente C is published. The radiocarbon dating and isotope analyses undertaken as part of the present research have contributed important information for reconstructing the possible number of humans buried within the deposits explored at Grotta d’Oriente, as will be discussed below.

## Methods

The human and faunal skeletal remains which are the object of the present study are stored at the Museo Archeologico Regionale “Antonino Salinas” in Palermo (Italy) and official permission was obtained from this institution to access, sample and loan the materials for destructive analyses.

### Palaeogenetic Analyses

Several recent ancient DNA (aDNA) studies have provided new insights into evolutionary changes in past human populations [35]–[36]. Despite the enormous potential offered by palaeogenetic analyses and the notable advances that have been accomplished, there are methodological constraints which are still difficult to overcome. In fact, with the exception of permafrost samples that contain endogenous DNA in higher relative abundance, DNA molecules are generally not well preserved and are highly damaged in ancient samples [37]. Depending on the state of preservation of the material, experimental artefacts are, thus, possible or even likely outcomes of palaeogenetic analyses on prehistoric remains. Nucleotide misincorporations by DNA polymerases during amplification, which may be induced by chemical modifications in the ancient DNA, are one source of artefacts [37]–[38]. Contamination by modern DNA, which may be introduced during handling of the specimen or subsequent laboratory procedures, represents another source of artefacts in aDNA analyses [39]. This is more of a problem with remains of modern humans, given that modern contaminating molecules can be misinterpreted as endogenous aDNA [37]. Provided strict measures of authentication are taken, however, the recovery of reliable ancient human DNA is indeed possible [40]–[43].

The genetic background of humans from Sicily, as inferred by molecular markers, is still open to debate [44]–[45], because as other Mediterranean islands it has had a complex demographic history reflecting the influx of migrating people of Mediterranean and Middle Eastern ancestry. In addition, no evidence is available on the genetic origin of the humans that first peopled the island. The results of the analyses of the hypervariable region I (HVR-1) of the mitochondrial DNA (mtDNA) of individual Oriente B, reported here, provide data on the aDNA of the possible descendants of the humans that were responsible for the definitive colonization of Sicily.

#### Ancient DNA precautions

The tenth lumbar vertebra of individual Oriente B was selected for the present study because it was thickly encrusted by deposit and had not been studied yet by the physical anthropologists. This allowed us to exclude the possibility of superficial contamination by exogenous DNA through handling at the excavation stage. Any remaining contaminating exogenous DNA was removed during the cleaning step of the DNA extraction step. The vertebra was sampled using disposable gloves and face masks to prevent contamination from handling, after which the bone sub-samples were placed in sterile tubes. The palaeogenetic analyses were conducted in two laboratories exclusively dedicated to aDNA work, at the University of Florence and at the Pompeu Fabra University of Barcelona. All steps of the analyses were replicated at least twice in each laboratory and multiple measurements were done to exclude contamination and potential artifactual changes (including post-mortem damage). Disposable masks, gloves, and laboratory coats were worn during the experiments and were changed frequently. Racks, pipettors and containers were treated with undiluted bleach. Exposed surfaces were wiped down with bleach and UV irradiated before each procedure. In each set of extractions or amplifications, we included a negative control, represented by all the reagents except the bone powder, and these negative controls, together with blanks (all amplification reagents minus DNA), were regularly analysed in every PCR experiment to control for presence of exogenous DNA. Each PCR product was then cloned and the sequence determined from multiple clones. To test for possible contamination within the Florence laboratory, a sub-sample of Oriente B was subject to DNA extraction, amplification, cloning and sequencing in Barcelona. Furthermore, all aDNA sequences obtained were compared with the HVR-1 sequences of the researchers who handled the specimen at the Museo Archeologico Regionale “Antonino Salinas” prior to sampling and in the aDNA laboratories, in order to exclude the possibility that they had been affected by recent contamination.

#### DNA extraction

All preparation and extraction methods followed strict protocols for aDNA analyses [37]. Extraction procedures were performed in a pre-PCR area exclusively dedicated to palaeogenetic analyses with positive air pressure and overnight UV light, physically isolated from those in which PCR cyclings and post-PCR analysis are conducted.

The outer layer of the lumbar vertebra of Oriente B was removed with a rotary tool and the resulting fragment was then soaked in 10% bleach. After brushing and soaking the bone was irradiated for an hour under UV light and powdered. In Florence, DNA was extracted following a silica-based protocol [40]. In Barcelona the extraction was performed using a protocol described by Sampietro et al. [46]. At least two independent extracts were obtained from each sub-sample and multiple negative controls were included in each extraction.

#### DNA amplification

Two microliters of DNA extracted from the bone were amplified with this profile: 50 cycles of PCR (denaturation, 94°C for 45 s, annealing, 53°C for 1 min and extension, 72°C for 1 min) and final step at 72°C for 10 min. The 50-µl reaction mix contained 2 U of AmpliTaq Gold (Applied Biosystems), 200 µM of each dNTP and 1 µM of each primer. The 360-bp long HVR-1 was subdivided in three overlapping fragments using the following primer pairs: L15,995/H16,132; L16,107/H16,261; L16,247/H16,402 [40]. Each extract was amplified at least twice. Since overlapping primers were used throughout the PCR amplifications, it is highly unlikely that we amplified a nuclear insertion rather than the organellar mtDNA. The protocol of the amplifications conducted in the Barcelona laboratory is described by Sampietro et al. [46].

#### Cloning and sequencing

In both laboratories, PCR products were cloned using the TOPO TA Cloning Kit (Invitrogen) following the manufacturer’s instructions. White recombinant colonies were screened by PCR, transferring the colonies into a 30-µl reaction mix [67 mMTris–HCl (pH 8.8), 2 mM MgCl2, 1 µM of each primer, 0.125 mM of each dNTP, 0.75 U of Taq Polymerase] containing M13 forward and reverse universal primers. After 5 min at 92°C, 30 cycles of PCR (30 s at 90°C, 1 min at 50°C, 1 min at 72°C) were carried out and clones with insert of the expected size were identified by agarose gelelectrophoresis. After purification of these PCR products with Microcon PCR devices (Amicon), a volume of 1.5 µl was cycle-sequenced following the BigDye Terminator kit (Applied Biosystems) supplier’s instructions. The sequence was determined using an Applied BioSystems 3100 DNA sequencer.

### AMS Radiocarbon Dating on Human Bone Collagen

The skeletal remains of three humans unearthed in the course of the 1972 excavations (Oriente A, B and X) have been sampled for AMS radiocarbon dating. Collagen was extracted and prepared for AMS radiocarbon dating according to the bone pre-treatment methods devised by Talamo and Richards [47]. Oriente A (S-EVA 2802) was not dated because the mandible of this individual did not yield collagen. The radiocarbon dates for Oriente B and × (Table 3) were calibrated with OxCal 4.1. [48] using the IntCal09 calibration curve [49].

**Table 3:** AMS radiocarbon dates on the bone collagen of the two Mesolithic individuals.

Max Planck Institute laboratorynumber	AMS radiocarbon laboratory number	Individual	^14^C Date (BP)	Calendar age cal. BC (2σ)	Calendar age cal. BP (2σ)
S-EVA 8378	OxA-V-2364-37	Oriente X	8653±39	7750 (p. 95.4%) 7580	9690 (p. 95.4%) 9530
S-EVA 2799	KIA-36049	Oriente B	9275±45	8630 (p. 95.4%) 8340	10580 (p.95.4%) 10290
S-EVA 2800	KIA-36050	Oriente B	9395±45	8790 (p. 95.4%) 8560	10740 (p. 95.4%) 10510
S-EVA 2801	KIA-36051	Oriente B	9440±40	8840 (p. 95.4%) 8610	10790 (p. 95.4%) 10560
Modelled date combining		Oriente B	9377±25	8734 (p. 91.2%) 8595	10683 (p. 91.2%) 10544
S-EVA 2799, 2800, 2801				8589 (p. 4.2%) 8570	10538 (p. 4.2%) 10519

The dates were performed at the Oxford Radiocarbon Accelerator Unit and at the Liebniz Laboratory of the Christian Albrechts Universität of Kiel on the bone collagen of two Mesolithic humans from Grotta d’Oriente. The dates were calibrated with Oxcal 4.1. [48] using the IntCal09 calibration curve [49]. The date on sample S-EVA 2800 (KIA-36050) had previously been reported by D’Amore et al. [67]. The age obtained by modelling the AMS radiocarbon dates from the three rib fragments of Oriente B (S-EVA 2799-2801), however, represents a more accurate estimate of the chronology of this individual. The calibration of the ^14^C dates done by taking into account the potential estimated proportions of marine foods consumed by Oriente × (<20%) and Oriente B (<10%), and the marine reservoir correction for Sicily [82], produces respectively calendar ages of 9620 (p.95.4%) 9480 cal. BP and 10580 (95.4%) 10420 cal. BP. These age ranges are only marginally more recent than those shown in the table, calculated assuming fully terrestrial diets.

### Carbon and Nitrogen Isotope Analyses on Human and Animal Bone Collagen

Collagen extraction for carbon and nitrogen isotope analyses was performed following protocols proposed by Brown et al. [50] and modified by Richards and Hedges [12]. The carbon and nitrogen isotope analyses were done at the Max Planck Institute for Evolutionary Anthropology (Leipzig, Germany) on a Thermo Finnigan Flash EA coupled to a Delta Plus XP isotope ratio monitoring mass spectrometer. The δ^13^C values are reported relative to the V-PDB standard and the δ^15^N values are reported relative to the AIR standard. The analytical precision based on repeated measurements was <0.20‰.

The five human bones from Oriente A (mandible), Oriente B (3 ribs) and Oriente × (ulna) sampled for AMS radiocarbon dating were also sampled for carbon and nitrogen isotope analyses (Tables 3 and 4). All these bones, with the exception of the mandible of Oriente A (S-EVA 2802) that did not conserve its organic fraction, yielded extracts with C/N molar ratios compatible with those of collagen [51]. The yields from the three ribs sampled from Oriente B are all high (>3.1%), while the yield from Oriente × is lower (0.9%), but close to the 1.0% minimal weight percentage recommended by van Klinken [52] for well-preserved collagen. Taken together the different measures of quality control (%C, %N, C/N and %yield) indicate that the extracts from the human bone samples are all well-preserved collagen and can be validly used for AMS dating and palaeodietary analyses. Fourteen animal bones of the main species present in Upper Palaeolithic and Mesolithic deposits (*Cervus elaphus*, *Bos primigenius*, *Sus scrofa*, *Vulpes vulpes* and *Epinephelus marginatus*) and four from the late Mesolithic and Mesolithic-Neolithic deposits (*Ovis vel Capra* and *Epinephelus marginatus*) were sampled. The %C, %N, C/N molar ratios and yields of the extracts from the sixteen bones that yielded an extract are compatible with collagen (Table 4) and can, therefore, be used for the present study according to the criteria described by van Klinken [52].

**Table 4:** Carbon and nitrogen isotope values from bone collagen of the prehistoric humans and fauna from Grotta d’Oriente.

Lab. N. (S-EVA)	Context	Species	Bone	δ^13^C (‰)	δ^15^N (‰)	%C	%N	C/N	% coll.
8378^*^	Oriente X	*Homo sapiens*	Ulna	−17.8	10.6	38.9	14.2	3.2	0.9
2799	Oriente B	*Homo sapiens*	Rib	−18.9	11.1	43.5	15.7	3.2	3.1
2800	Oriente B	*Homo sapiens*	Rib	−18.8	11.2	43.3	15.4	3.3	3.8
2801	Oriente B	*Homo sapiens*	Rib	−19.0	11.6	43.8	15.8	3.2	5.3
13802	C110/180	*Cervus elaphus*	Metacarpal	−20.7	8.5	43.1	15.0	3.4	6.2
13803	B114/160	*Cervus elaphus*	Phalanx I	−20.5	5.8	43.0	15.0	3.3	6.9
13807	B80/100	*Cervus elaphus*	Phalanx II	−21.0	5.9	41.8	14.6	3.3	3.2
13812	B60/80	*Bos primigenius*	Phalanx II	−21.0	6.7	42.4	14.7	3.4	5.1
13814^*^	B60/80	*Bos primigenius*	Ischium	−21.2	6.8	45.4	16.3	3.3	12.1
13810	C75/110	*Sus scrofa*	Radius	−20.1	8.4	43.7	15.4	3.3	5.9
13811	C75/110	*Sus scrofa*	Metapodial	−21.8	4.2	43.4	15.3	3.3	12.4
13804	B80/100	*Vulpes vulpes*	Humerus	−20.0	7.3	43.0	15.1	3.3	5.1
13805	B100/114	*Vulpes vulpes*	Ulna	−19.5	7.8	42.3	14.7	3.4	2.3
13813	B60/80	*Vulpes vulpes*	Humerus	−19.5	7.0	43.5	15.3	3.3	8.7
13798	B40/60	*E. marginatus*	Quadratum	−8.9	9.3	30.1	10.6	3.3	3.5
13799	B40/60	*E. marginatus*	Dentary	−9.6	9.3	42.9	14.9	3.3	4.6
13800	C14/40	*E. marginatus*	Frontal	−9.7	9.9	42.0	14.8	3.3	5.1
13808	A80/114	*Ovis vel Capra*	Femur	−21.7	6.6	43.9	15.0	3.4	8.6
13809	A80/114	*Ovis vel Capra*	Radius	−16.2	6.3	44.3	15.9	3.3	13.8
13815	C14/40	*Ovis vel Capra*	Mandible	−20.4	7.5	43.5	15.1	3.4	9.9

The fauna includes: red deer (*C. elaphus*), aurochs (*B. primigenius*), wild boar (*S. scrofa*), red fox (*V. vulpes*), ovicaprids (*Ovis vel Capra*) and dusky grouper (*E. marginatus*). The *C. elaphus* specimen S-EVA 13802 originates from stratigraphic unit 110–180 cm in Trench C and is in a different state of preservation compared to all other bones analyzed, thereby pre-dating the Mesolithic humans (Oriente B and X) and most certainly also the late Upper Palaeolithic individual (Oriente C). The wild boar (*S. scrofa*) specimen S-EVA 13810 is a juvenile and its high δ^15^N value compared to S-EVA 13811 (an adult) probably reflects in part the isotopic composition of protein acquired before weaning. The samples of ovicaprids (*Ovis vel Capra*) are early Neolithic and post-date all the humans from Grotta d’Oriente. This might also be the case of the dusky grouper (*Epinephelus marginatus*) specimen from stratigraphic unit 14–40 cm of Trench C (S-EVA 13800).

## Results

### Palaeogenetic Analyses

A reproducible HVR-1 sequence between positions 16,024 and 16,384 of the reference sequence reported in Table S1 (Supporting Information) was obtained from the vertebra of Oriente B [53]. All extraction and PCR blanks were consistently negative throughout the study, indicating that the results are unlikely to derive from contaminants in the extraction or PCR processes. To detect possible nucleotide misincorporations during the amplification reactions, which could affect most of the molecules of the amplified products, at least two independent PCR reactions were performed for each DNA extract. Despite cloned sequences showed evidence of post-mortem damage, derived miscoding lesions that are typical for ancient DNA [38] and the diagnostic substitution 16067T in all clones were observed. According to the haplogroup nomenclature [54]–[55], the distinctive transition in the Oriente B sample places the sequence within haplogroup HV-1. The mtDNA sequences of those who came into contact with the analysed specimen do not match that of Oriente B and this makes the possibility of any contamination by modern DNA highly unlikely (Table 5). The result of our palaeogenetic study is important because it represents the first mtDNA data available for an early Holocene (Mesolithic) human from southern Europe or the Mediterranean. In addition, the fact that the mtDNA of Oriente B belongs to the HV-1 haplotype is noteworthy, because it has been suggested that most of the HV haplogroups in Europe expanded from the Near East and Caucasus region before the Last Glacial Maximum, having a coalescence age of 30,000±4,000 BP [54]–[56]. The HV-1 haplogroup, which belongs to the HV-family, is absent in most of Europe and India and it seems to have an epicentre of frequency and diversity in the Trans-Caucasus area. This suggests that the ancestors of the hunter-gatherers of Favignana might ultimately have originated from the Near East and Caucasus region.

**Table 5:** Mitochondrial HVR-1 haplotypes of the researchers that have been in physical contact with the vertebra of Oriente B.

Researcher	Task	HVR-1 haplotype
Giulio Catalano	palaeogenetic analysis	16069T 16126C 16261T 16311C
Carles Lalueza-Fox	palaeogenetic analysis	16126C 16294T 16296T 16304C
David Caramelli	palaeogenetic analysis	16193T 16278T
Andrea Messina	anthropological study	16129A 16223T 16391A
Luca Sineo	anthropological study	16076T 16093C

### AMS Radiocarbon Dating

Of the three individuals sampled, two yielded collagen suitable for AMS radiocarbon dating (Table 3). Oriente B dates to 9,377±25 ^14^C BP, which is the modelled date estimated with OxCal 4.1. using the AMS radiocarbon dates on the three ribs from this individual (S-EVA 2799, 2800 and 2801). The cumulative range of the calibrated ages from the three dates on the ribs of Oriente B is 10,790-10,290 cal BP, while the calibrated age based on the modelled date combining the dates for the three samples from Oriente B is 10,680-10,520 cal BP. This individual can, therefore, be assigned to the beginning of the Holocene (Preboreal; immediately after the Younger Dryas stadial), confirming its attribution, based on stratigraphic and archaeological observations, to the early Mesolithic.

The ulna (S-EVA 8378) has been dated to 8,653±39 ^14^C BP, which is equivalent to a calibrated age of 9,690-9,530 cal BP. This radiocarbon date assigns this individual also to the early Holocene (Boreal), implying that the bone in question belongs to a Mesolithic hunter-gatherer. As mentioned above, the ulna cannot belong to Oriente C on anatomical grounds and the date is further evidence for this, given that this individual is thought to date back to three millennia earlier [22]. If the ulna belongs to Oriente A, then this individual was also Mesolithic and not late Upper Palaeolithic, as suggested on the basis of stratigraphic observations made during the two excavation campaigns at the cave. The similarity between the sets of ornamental shells discovered with Oriente A and B might, in fact, be indicative of a strong cultural affinity, which could argue for the Mesolithic age of both burials. If the reconstructions of the stratigraphy of the deposits at Grotta d’Oriente are correct, then it can be concluded that Oriente A was disturbed by funerary or other activities performed by the Mesolithic occupants of the cave. In the impossibility of dating Oriente A, this issue remains unsolved and we maintain the attribution of the ulna to Oriente X. In conclusion, the radiocarbon dates on Oriente B and Oriente × testify that Grotta d’Oriente was used as a burial site during the early Mesolithic.

### Carbon and Nitrogen Isotope Analyses

**Figure 3:**
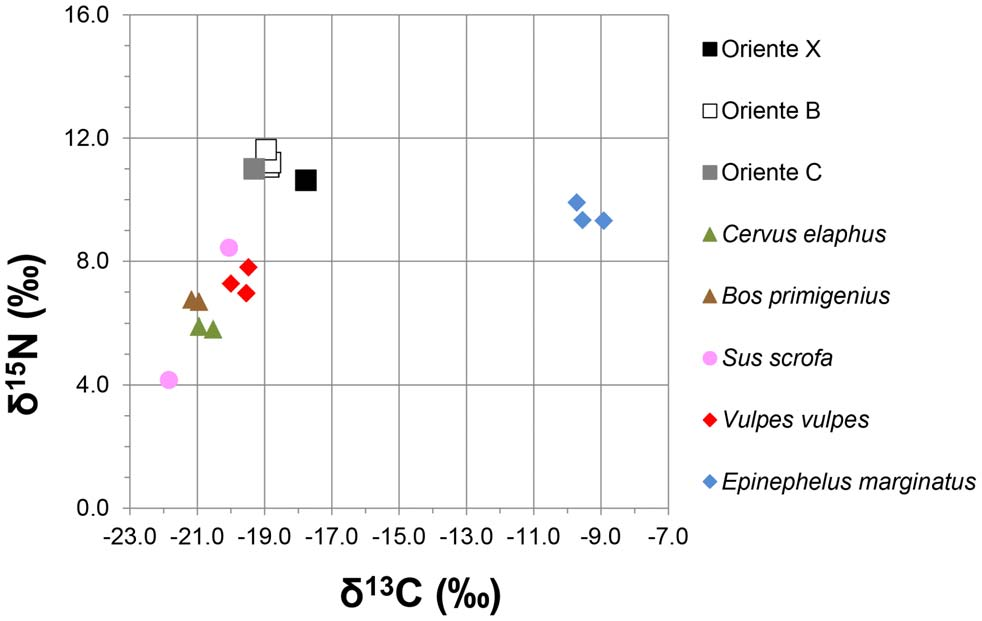
Carbon and nitrogen isotope composition of bone collagen from Mesolithic humans and fauna of Grotta d’Oriente.

The δ^13^C values of the collagen extracted from the late Pleistocene and early Holocene terrestrial animal bones from Grotta d’Oriente fall within a range of 2.3‰, between −21.8‰ and −19.5‰ (Table 4; Fig. 3). These results indicate that herbivores were feeding on C_3_ plants and that carnivores were feeding on terrestrial herbivores living in a C_3_ ecosystem, which is consistent with the environments of Sicily and of the European shores of the Mediterranean. Dusky grouper (*E. marginatus*) collagen yielded δ^13^C values from −9.7‰ to −8.9‰ (mean = −9.4±0.4‰) and δ^15^N values from 9.3‰ to 9.9‰ (mean = 9.5±0.3‰), compatible with the diet of a coastal carnivorous fish [57]. The δ^13^C values of the human bone collagen are −18.9‰ for Oriente B and −17.8‰ for Oriente X. The mean δ^13^C value for terrestrial mammals is −20.5±0.8‰, while the mean δ^13^C value for marine fish is −9.4±0.4‰. Taking these means as end members in a linear mixing model, and allowing for a mean offset between prey and predator of 0.5‰, it can be assumed (as done in other studies to obtain rough approximations of the proportion of terrestrial versus marine protein consumed [12], [58]) that individuals with δ^13^C values of circa −20.0‰ had entirely terrestrial diets, while those with δ^13^C values of circa −9.0‰ had entirely marine diets. In most cases, an increase in δ^13^C values of circa 1.0‰ equates to an increase in marine food intake of roughly 10% [59]. Linear interpolations based on the δ^13^C values of Oriente B and Oriente X, using the end members above, suggest that these humans may have consumed respectively <10% and <20% marine protein originating from coastal fish such as grouper, with the rest of the dietary protein coming from terrestrial foods. Vika and Theodoropoulou [60] claim that the δ^13^C values of Mediterranean marine fish overlap with those of terrestrial fauna and, hence, that fish consumption may not be detectable. The most extreme example of this is offered by one of the Serranidae (i.e. sea basses and groupers) from the Mesolithic site of Cyclops cave on the island of Youra (Greece). These fish are fully marine, but despite this the isotopic composition of this individual falls outside the marine range (δ^13^C = −19.2‰; δ^15^N = 8.9‰). Isotope analyses on bone collagen from other Serranidae by Vika and Theodoropoulou [60], as well as in the present and other studies [57], confirm that Mediterranean species of this family have fully marine diets. The anomalous isotopic composition of the Serranidae from Youra might be due to incorrect taxonomic identification or to poor collagen preservation, despite the fact that this specimen just meets the quality criteria. Other overlaps noted by Vika and Theodoropoulou [60] might be due to the fact that the fish were only identified to family and, therefore, the habitat classification attributed to each specimen might not be correct at the species level, because the families in question (e.g. Sparidae) include taxa that live in aquatic environments characterized by different salinity regimes (i.e. freshwater, euryhaline or marine), and in combinations of these at different ontogenic stages. It should also be noted that studies on living fish from the Mediterranean have not demonstrated anomalous overlaps in isotopic composition with the terrestrial biome [61]–[62]. Vika and Theodoropulou [60] correctly point out, however, that the consumption of brackish and freshwater fish might be difficult to establish and doing so requires interpreting the isotopic data with the rest of the archaeological evidence, as will be done below for Grotta d’Oriente.

The δ^15^N values are 11.3 ‰ for Oriente B and 10.6‰ for Oriente X, which are respectively 5.0‰ and 4.3‰ higher than the mean for herbivores and omnivores (6.3±1.4‰), and 3.9‰ and 3.2‰ higher than the mean (7.4±0.4‰) for fox (*Vulpes vulpes*), the only carnivore in the assemblage. S-EVA 13802 is a *C. elaphus* specimen from Layer 110–180 cm in Trench C and thereby pre-dates the Mesolithic (Oriente B and X) and the late Upper Palaeolithic (Oriente C) humans. This bone is in a different state of preservation compared to all others analyzed and its high δ^15^N ratio (8.5‰) is probably due to the presence of different, possibly drier, climatic conditions during its lifetime.

Given that the widest estimate for the trophic level shift from a carnivore to its prey is 3–5‰ [63], these data indicate that the humans in question had diets characterized by high levels of meat consumption, with terrestrial herbivores (e.g. *C. elaphus* and *B. primigenius*) and omnivores (e.g. *S. scrofa*) constituting the most frequent prey.

The present analysis has not included the isotopic data from the bone collagen of the ovicaprids (*Ovis vel Capra*), because these animals are early Neolithic and, hence, not contemporary to the humans. It should, however, be noted that one of these specimens (S-EVA 13809) has a δ^13^C value (−16.2‰) that is noticeably higher than those of the other two ovicaprids analysed and of the wild terrestrial fauna from Grotta d’Oriente (mean = −20.5±0.8‰). The carbon isotope composition of S-EVA 13809 suggests that part of the dietary protein consumed by this ovicaprid originated from C_4_ plants, which were not eaten by any other terrestrial herbivore. One possible explanation for this is that the individual in question was imported from regions where C_4_ plants are readily available, such as North Africa or the Near East, an intriguing hypothesis for the study of the spread of the Neolithic economy, which should be explored further.

## Discussion

The results of the analyses on the human and faunal remains from Grotta d’Oriente presented here provide new insights on the origin, culture, diet and subsistence of the hunter-gatherers that inhabited Sicily and the area of the Ègadi Islands during the Late Pleistocene and early Holocene.

### Origin and Culture

The palaeogenetic data presented in this paper has important implications for our understanding of the peopling of Sicily by Anatomically Modern Humans (AMH). At present, we have almost no palaeogenetic data for Upper Palaeolithic and Mesolithic hunter-gatherers from southern Europe and the Mediterranean. A notable exception is represented by one of the Upper Palaeolithic humans from Grotta Paglicci in south-eastern Italy, which belongs to either haplogroup HV or pre-HV [64]. This individual was part of groups of hunter-gatherers that were the makers of Gravettian and, after the Last Glacial Maximum, Epigravettian lithic industries [65]. The first undisputed colonization of Sicily has been linked to Epigravettian groups that must have crossed what is now the Strait of Messina sometime after the Last Glacial Maximum, given that no stratified site on the island dates to before that time [8], [28], [66]. The attribution of individual Oriente B to the HV-1 haplogroup might, therefore, tentatively be interpreted as further evidence in favour of the descendance of the early Holocene hunter-gatherers of Sicily, as well as those from Favignana, from the human groups that occupied southern Italy before (Gravettian) and after (Epigravettian) the Last Glacial Maximum [64]–[65]. This is in line with the results of a morphometric study on the cranium of Oriente B [67], which showed that it is morphologically similar to middle and late Upper Palaeolithic (i.e. Gravettian and Epigravettian) humans from the Italian Peninsula and continental Europe, as well as to Mesolithic humans from southern Germany and northern Europe. The few sources of evidence currently at our disposal suggest that groups of Upper Palaeolithic humans probably migrated to Sicily at the same time as the mammals of the Late Pleistocene faunal complex of Castello [68], as recently hypothsized by a morphometric study on the Epigravettian humans buried at Grotta di San Teodoro in NE Sicily [69]. This complex dates to around the Last Glacial Maximum and includes species such as *Equus hydruntinus* (European ass) that would have required a land bridge connection in order to reach Sicily [70]. Our findings could also imply that AMH reached Sicily relatively late in the Upper Palaeolithic, casting further doubt on the attribution of Riparo di Fontana Nuova to the Aurignacian culture [71], a claim unsupported by absolute dating and disputed on typological grounds [8], [28]. The scenario we propose is a working hypothesis, given that only further palaeogenetic studies can confirm whether HV haplogroups are representative of those of the first AMH that migrated to Sicily.

The AMS radiocarbon dates on Oriente B and on another human buried at Grotta d’Oriente (Oriente X) demonstrate that this cave was used as a burial site during the Mesolithic. The fact that Oriente C might date to the late Upper Palaeolithic [22] could indicate the use of the cave for funerary purposes also towards the beginning of its occupation. The burials unearthed at Grotta d’Oriente seem to share many features with those of late Upper Palaeolithic (Epigravettian) tradition from Italy and Sicily, given that they are single primary depositions, interred in shallow graves in a variety of positions, depending on the shape of the pit, and contained few or no grave goods [72].

An aspect of the burial customs at Grotta d’Oriente that sets it apart from contemporary prehistoric sites is the inclusion of sets of worked shells in Oriente A and Oriente B [26], [34]. These beads and pendants were either part of necklaces, as hypothesized by Mannino [23], or of other body ornaments, which have not been found in any other contemporary site in Sicily or Italy. Did this apparent variance in funerary customs result from the development of intra-cultural diversity between different Mesolithic groups living in Sicily? The data at hand, which can inform us on this question, suggest that this might indeed have been the case. As discussed by Mannino and Thomas [73], archaeological evidence of changes in aspects of hunter-gatherer lifeways as art and subsistence strategies supports the notion that new territorial divisions had developed in western Sicily by the late Mesolithic, culminating in the establishment of well-defined territories. In a study of burial practices in the Italian Peninsula and Sicily, from the middle Upper Palaeolithic to the Mesolithic, Gazzoni and Fontana [72] point out that the most significant changes in funerary customs took place in the Late Glacial and early Holocene, probably as a result of social and territorial rearrangements, which might ultimately have been caused by environmentally-driven changes in subsistence strategies. The changes in the funerary customs of the hunter-gatherers of the Ègadi Islands might be linked to the upheavals resulting from environmental changes, such as those caused by rising sea levels in the early Holocene. In the thousand-year time interval separating Oriente B and Oriente X, sea level rose around 0.4 m every twenty years (based on the curve by Lambeck et al. [20]), which is fast enough for it to have been noticed within a lifetime. These dramatic changes must have affected the perception of the world and symbolic sphere of these hunter-gatherers well before they forced the adoption of new subsistence strategies, which, as we shall see in the next section, did not change significantly as the Ègadi were becoming islands.

### Diet and Subsistence

The faunal assemblages from late Upper Palaeolithic and Mesolithic sites of Sicily testify that there was little change in the subsistence of the hunter-gatherers that populated the island in the closing stages of the Pleistocene and in the first two thousand years of the Holocene [4], [25], [27]–[28], [73]. Hunting of terrestrial mammals, such as red deer, European ass and aurochs, was still the main subsistence activity right up to the early Holocene [4], although a range of other foods, including coastal resources, were undoubtedly consumed [27]–[28], [74]. These, however, were often mainly terrestrial and marine molluscs, with fishing and wildfowling constituting secondary activities until the late Mesolithic, when, during the so-called Mesolithic-Neolithic ‘transition phase’, the exploitation of marine resources increased and, for a brief period, possibly even included the meat of stranded cetaceans [4].

In order to establish whether the dearth of evidence for the exploitation of aquatic resources, and particularly marine fish, is truly representative of the importance of such foods in the diets of the Mesolithic hunter-gatherers of western Sicily we undertook carbon and nitrogen isotope analyses. The individuals from Grotta d’Oriente are potentially very useful in this respect, given that they lived at a time when the Ègadi Islands were being isolated by rising sea levels and their territory was becoming increasingly maritime and marginal for the exploitation of terrestrial resources. The results of these analyses, nevertheless, show that seafood contributed generally a small proportion of the dietary protein consumed by the humans in question, in line with the palaeodietary reconstructions based on the study of the faunal remains recovered at Grotta d’Oriente [26], [28] and at other early Mesolithic sites in NW Sicily [4].

In fact, whilst the higher δ^13^C value of the later Mesolithic individual (Oriente X) could be due to a slightly higher consumption of low trophic level marine (possibly brackish) taxa, such a difference may even be mainly down to inter-individual variability and thus not significant given that low amounts of marine protein are not detectable [59]. The late Upper Palaeolithic individual unearthed during the excavations of 2005, Oriente C, analyzed by Craig et al. [14], has similar δ^13^C ( = −19.3‰) and δ^15^N ( = 11.0‰) values to the two Mesolithic individuals (Oriente B and Oriente X). The difference between the δ^13^C values of Oriente C and Oriente × suggests that these are different individuals, confirming the observations based on the anatomical features and on the dating. The δ^13^C value of Oriente C is lower than both Oriente B ( = −18.9‰) and Oriente × ( = −17.8‰), which again could suggest that there was a progressive, albeit not very substantial, increase through time in the exploitation of marine resources at Grotta d’Oriente. An increase in the δ^13^C values can also be caused by a reduction in tree cover (‘canopy effect’), but there is no evidence for this at the time in question [75]. On the contrary, oxygen isotope analyses on the skeletal carbonates of terrestrial gastropods (*Eobania vermiculata*) from Grotta d’Oriente suggest that there was an increase in rainfall in the transition from the Pleistocene to the early Holocene, which in theory should have favoured the spread of arboreal vegetation [31]. It is, therefore, unlikely that the increase in the δ^13^C values of the bone collagen of the three humans from Grotta d’Oriente was the result of a climatically-driven environmental change and a dietary transition, hence, seems to be the most likely explanation. The magnitude of this change, however, would have been minor, given that the values shift in total by a mere 1.5‰ and remain within the range of an essentially terrestrial diet. On the basis of the isotopic data at hand, following Vika and Theodoropoulou’s [60] observations, it is also not possible to establish to what extent, if at all, this shift was due to the consumption of freshwater and euryhaline species. The scarce evidence for the exploitation of such taxa and the fact that rising sea levels submerged low-lying coasts, which were probably characterized by brackish and freshwater habitats [27]–[28], imply that it is unlikely that the increase in δ^13^C values reflects a reliance on resources from such environments.

The low-level consumption of marine foods by Oriente B and C is analogous to that verified, through isotope analyses, on other late Upper Palaeolithic and Mesolithic humans from Sicily [5], [8]–[9] and most areas of the Mediterranean [5]–[6]. On the other hand, the slightly higher δ^13^C ratio of Oriente × is similar to those of a few individuals from El Collado (Spain), Vela Spilja – Vela Luka (Croatia) and Corsica, who acquired about a fifth or more of their protein from the sea [7], [10]–[11]. Palaeodietary studies also show that marine resources only contributed a minor amount of food to the diets of later prehistoric and historic period Mediterranean people [76], in spite of having more advanced fishing technologies than prehistoric hunter-gatherers [77]. As proposed in most of the above-mentioned isotopic studies, the lower reliance on marine resources by prehistoric hunter-gatherers in the Mediterranean, compared to their contemporaries living on the Atlantic coasts of Europe, is chiefly due to the oligotrophy of this quasi-enclosed sea [16] and of its coastal habitats [15]. On the Atlantic coasts of western and northern Europe, the high primary productivity of the ocean supported a secondary biomass that allowed Late Glacial and Postglacial hunter-gatherers to intensify food acquisition by exploiting marine resources [12]–[13]. As predicted by Binford’s [78] terrestrial model for mid-latitude regions, Mediterranean hunter-gatherers also broadened their subsistence base from exploiting mainly terrestrial, high-ranking, resources, such as wild mammals, to preying on lower-ranking animals, including marine fauna [2]. However, the isotopic data presented here, the lack of archaeological evidence for a fishing technology which was sufficiently sophisticated to exploit intensively Mediterranean fisheries [79] and the scarcity of fish remains in late Pleistocene and early Holocene deposits of Sicily, all suggest that the hunter-gatherers of Grotta d’Oriente only relied to a limited extent on coastal and marine resources. These findings also imply that it is unlikely that sites submerged by Late Glacial and Postglacial sea level rise contain assemblages with proportions of marine faunal remains high enough to overturn this scenario [17].

### Conclusions

The palaeogenetic data obtained from Oriente B, a Mesolithic hunter-gatherer living on Favignana, represents the first mtDNA data available for an early Holocene human from Mediterranean Europe. Other lines of investigation undertaken as part of the present study show that, in spite of rising sea levels and progressive isolation, during the late Pleistocene – early Holocene transition the hunter-gatherers of Favignana maintained the subsistence strategies of the groups from which they descended. Coastal ecosystems changed from being characterized by a variety of different shore types, including transitional waters and lagoonal environments [27]–[28], to being progressively dominated by rocky shores and marginal for terrestrially-based hunting and gathering. As these changes were taking place, local hunter-gatherers did not develop strongly marine-oriented adaptations, such as those of their counterparts living on the Atlantic shores of Europe, but maintained essentially terrestrial-based strategies, similar to those of Late and Post-Glacial groups of the central and western Mediterranean (Table 1). This could be down to the continued availability of terrestrial resources or to the oligotrophy of the Mediterranean Sea. Alternatively, both these hypotheses might be valid, if human population levels were low. In fact, the lack of the development of a sophisticated fishing technology might not only be a consequence of the scarcity of its potential returns, but also of the small effective population size, which reduces the likelihood of invention [80], as well as the probability that fitness-enhancing innovations are effectively passed on [81]. These observations have important implications for the understanding of hunter-gatherer economies in the Mediterranean Basin during the Holocene and, consequently, of the shift to agro-pastoralism.

## Supporting Information

Table S1 **Clone sequences for the Oriente B sample.**
(DOC)

## References

[1] Colonese AC, Mannino MA, Bar-Yosef Mayer DE, Fa DA, Finlayson JC, et al (2011) Marine mollusc exploitation in Mediterranean prehistory: an overview. Quat Int 239: 86–103.

[2] Stiner MN, Munro N (2011) On the evolution of diet and landscape during the Upper Paleolithic through Mesolithic at Franchthi Cave (Peloponnese, Greece). J Hum Evol 60: 618–636. 21371735 10.1016/j.jhevol.2010.12.005

[3] Cortés-Sánchez M, Morales-Muñiz Simón-Vallejo MD, Bergadà-Zapata MM, Delgado-Huertas A, López-García P, et al (2008) Palaeoenvironmental and cultural dynamics of the coast of Málaga (Andalusia, Spain) during the Upper Pleistocene and early Holocene. Quat Sci Rev 27: 2176–2193.

[4] Tagliacozzo A (1993) Archeozoologia della Grotta dell’Uzzo, Sicilia. Da un’economia di caccia ad un’economia di pesca ed allevamento. Supplemento del Bullettino di Paletnologia Italiana 84. Rome: Poligrafico e Zecca dello Stato. 278 p.

[5] Francalacci P (1988) Comparison of archaeological trace element and stable isotope data from two Italian coastal sites. Rivista di Antropologia 56: 239–250.

[6] Paine C, O’Connell T, Miracle PT (2009) Stable isotopic reconstruction of Early Mesolithic diet at Pupićina Cave. In: McCartan S, Schulting R, Warren G, Woodman P, eds. Mesolithic Horizons. Oxford: Oxbow Books. 210–216.

[7] Lightfoot E, Boneva B, Miracle PT, Šlaus M, O’Connell TC (2011) Exploring the Mesolithic and Neolithic transition in Croatia through isotopic investigations. Antiquity 85: 73–86.

[8] Mannino MA, Di Salvo R, Schimmenti V, Di Patti C, Incarbona A, et al (2011) Upper Palaeolithic hunter-gatherer subsistence in Mediterranean coastal environments: an isotopic study of the diets of the oldest directly-dated humans from Sicily. J Archaeol Sci 38: 3094–3100.

[9] Mannino MA, Thomas KD, Leng MJ, Di Salvo R, Richards MP (2011) Stuck to the shore? Investigating prehistoric hunter-gatherer subsistence, mobility and territoriality in a Mediterranean environment through isotope analyses on marine mollusc shell carbonates and human bone collagen. Quat Int 244: 88–104.

[10] Garcia Guixé E, Richards MP, Eulàlia Subirà M (2006) Palaeodiets of humans and fauna at the Spanish Mesolithic site of El Collado. Curr Anthropol 47: 549–556.

[11] Vigne J-D (2004) Accumulation de lagomorphes et de rongeurs dans le sites mésolithiques corso-sardes: origines taphonomiques implications anthropologiques. In: Brugal JP, Desse J, eds. Petits animaux et sociétés humaines: du complément alimentaire aux ressources utilitaires. XXIVe rencontres internationales d’archéologie et d’histoire d’Antibes. Antibes: A.P.D.C.A. 261–281.

[12] Richards MP, Hedges REM (1999) Stable isotope evidence for similarities in the types of marine foods used by late Mesolithic humans at sites along the Atlantic coast of Europe. J Archaeol Sci 26: 717–722.

[13] Dupont C, Tresset A, Desse-Berset N, Gruet Y, Marchand G, et al (2009) Harvesting the seashores in the Late Mesolithic of Northwestern Europe: a view from Brittany. J World Prehist 22: 93–111.

[14] Craig OE, Biazzo M, Colonese AC, Di Giuseppe Z, Martinez-Labarga C, et al (2010) Stable isotope analysis of Late Upper Palaeolithic humans and fauna remains from Grotta del Romito (Cosenza), Italy. J Archaeol Sci 37: 2504–2512.

[15] Fa DA (2008) Effects of tidal amplitude on intertidal resource availability and dispersal pressure in prehistoric human coastal populations: the Mediterranean-Atlantic transition. Quat Sci Rev 27: 2194–2209.

[16] Siokou-Frangou I, Christaki U, Mazzocchi MG, Montresor M, Ribera d’Alcalá M, et al (2010) Plankton in the open Mediterranean Sea: a review. Biogeosciences 7: 1543–1586.

[17] Bailey GN, Flemming NC (2008) Archaeology of the continental shelf: marine resources, submerged landscapes and underwater archaeology. Quat Sci Rev 27: 2153–2165.

[18] Schoeninger MJ, DeNiro MJ (1984) Nitrogen and carbon isotopic composition of bone collagen from marine and terrestrial animals. Geochim Cosmochim Acta 48: 625–639.

[19] Antonioli F (1997) Problematiche relative alle variazioni recenti del livello del mare e sue interazioni con le comunità preistoriche in Sicilia. In: Tusa S, ed. Prima Sicilia: alle origini della società siciliana. Palermo: Ediprint. 146–155.

[20] Lambeck K, Antonioli A, Purcell A, Silenzi S (2004) Sea level change along the Italian coast for the past 10,000 yr. Quat Sci Rev 23: 1567–1598.

[21] Tusa S (1999) La Sicilia nella preistoria, second edition. Palermo: Sellerio editore. 720 p.

[22] Lo Vetro D, Martini F (2006) La nuova sepoltura epigravettiana di Grotta d’Oriente (Favignana, Trapani). In: Martini F, ed. La cultura del morire nelle società preistoriche italiane: studio interdisciplinare dei dati e loro trattamento informatico. Florence: Origines. 58–66.

[23] Mannino G (2004) La Grotta d’Oriente di Favignana (Egadi, Sicilia): risultati di un sondaggio esplorativo. Quaderni del Museo Archeologico Regionale “Antonino Salinas”. 8: 9–22.

[24] Agnesi V, Macaluso T, Orrù P, Ulzega A (1993) Paleogeografia dell’arcipelago delle Egadi (Sicilia) nel Pleistocene Superiore – Olocene. Il Naturalista Siciliano (Series 4) 17: 1–23.

[25] Cassoli PF, Tagliacozzo A (1982) La fauna della Grotta di Cala dei Genovesi a Levanzo. Rivista di Scienze Preistoriche 37: 48–58.

[26] Mannino MA, Thomas KD (2004) Studio archeozoologico dei reperti faunistici dalla Grotta d’Oriente a Favignana (Trapani). Quaderni del Museo Archeologico Regionale “Antonino Salinas”. 8: 23–54.

[27] Mannino MA, Thomas KD (2010) Studio preliminare del campione faunistico della Grotta Schiacciata a Levanzo (Trapani). Atti del 5°Convegno Nazionale di Archeozoologia. Rovereto (Trento): Edizioni Osiride. 97–99.

[28] Martini F, Lo Vetro D, Colonese AC, De Curtis O, Di Giuseppe Z, et al. (2007) L’Epigravettiano Finale in Sicilia. In: Martini F, ed. L’Italia tra 15000 e 10000 anni fa. Cosmopolitismo e regionalità nel Tardoglaciale, Atti della Tavola rotonda, Firenze 18 novembre 2005. Florence: Museo Fiorentino di Preistoria. 209–253.

[29] Bovio Marconi J (1952) Esplorazioni archeologiche a Levanzo e Favignana. Notizie degli Scavi di Antichità (Series VIII) 6: 185–199.

[30] Mannino MA, Thomas KD, Leng MJ, Piperno M, Tusa S, et al (2007) Marine resources in the Mesolithic and Neolithic at the Grotta dell’Uzzo (Sicily): evidence from isotope analyses of marine shells. Archaeometry 49: 117–133.

[31] Colonese AC, Zanchetta G, Drysdale RN, Fallick AE, Manganelli G, et al (2011) Stable isotope composition of Late Pleistocene-Holocene *Eobania vermiculata* (Müller, 1774) (Pulmonata, Stylommatophora) shells from the Central Mediterranean basin: data from Grotta d’Oriente (Favignana, Sicily). Quat Int 244: 76–87.

[32] Colonese AC, Troelstra S, Ziveri P, Martini F, Lo Vetro D, et al (2009) Mesolithic shellfish exploitation in SW Italy: seasonal evidence from the oxygen isotopic composition of *Osilinus turbinatus* shells. J Archaeol Sci 36: 1935–1944.

[33] Di Salvo R, D’Amore G, Mannino MA, Schimmenti V, Caramelli D, et al. (2008) Ecology, morphometry, and genetics of the Palaeo-Mesolithic human remains of Grotta d’Oriente, Favignana (Italy). Int J Anthropol (Special Issue): 273–277.

[34] Mannino MA, Thomas KD (2008) A research agenda for the archaeomalacological study of prehistoric human ecology in the coastal zone of NW Sicily. Archaeofauna 17: 35–45.

[35] Helgason A, Lalueza-Fox C, Ghosh S, Sigurðardóttir S, Sampietro ML, et al (2009) Sequences from first settlers reveal rapid evolution in Icelandic mtDNA pool. PLoS Genet 5 (1): e1000343. 10.1371/journal.pgen.1000343 PMC2613751 19148284

[36] Malmström H, Gilbert MTP, Thomas MG, Brandström M, Stora J, et al (2009) Ancient DNA reveals lack of continuity between Neolithic hunter-gatherers and contemporary Scandinavians. Curr Biol 19: 1758–1762. 19781941 10.1016/j.cub.2009.09.017

[37] Pääbo S, Poinar H, Serre D, Jaenicke-Despres V, Hebler J, et al (2004) Genetic analyses from ancient DNA. Ann Rev Genet 38: 645–679. 15568989 10.1146/annurev.genet.37.110801.143214

[38] Gilbert MTP, Willerslev E, Hansen AJ, Barnes I, Rudbeck L, et al (2003) Distribution patterns of postmortem damage in human mitochondrial DNA. Am J Hum Genet 72: 32–47. 12489041 10.1086/345378 PMC420011

[39] Sampietro ML, Gilbert MTP, Lao O, Caramelli D, Lari M, et al (2006) Tracking down human contamination in ancient human teeth. Mol Biol Evol 23: 1801–1807. 16809622 10.1093/molbev/msl047

[40] Caramelli D, Vernesi C, Sanna S, Sampietro L, Lari M, et al (2007) Genetic variation in prehistoric Sardinia. Hum Genet 122: 327–336. 17629747 10.1007/s00439-007-0403-6

[41] Cooper A, Poinar HN (2000) Ancient DNA: do it right or not at all. Science 289: 1139. 10970224 10.1126/science.289.5482.1139b

[42] Hofreiter M, Serre D, Poinar HN, Kuch M, Pääbo S (2001) Ancient DNA. Nat Rev Gen 2: 353–359. 10.1038/35072071 11331901

[43] Rizzi E, Lari M, Gigli E, De Bellis G, Caramelli D (2012) Ancient DNA studies: new perspectives on old samples. Genet Sel Evol 44: 1–21. 22697611 10.1186/1297-9686-44-21 PMC3390907

[44] Rickards O, Martinez-Labarga C, Scano G, De Stefano GF, Biondi G, et al (1998) Genetic history of the population of Sicily. Hum Biol 70: 699–714. 9686481

[45] Francalacci P, Morelli L, Underhill PA, Lillie AS, Passarino G, et al (2003) Peopling of three Mediterranean Islands (Corsica, Sardinia, and Sicily) inferred by Y-chromosome biallelic variability. Am J Phys Anthropol 121: 270–279. 12772214 10.1002/ajpa.10265

[46] Sampietro ML, Lao O, Caramelli D, Lari M, Pou R, et al (2007) Paleogenetic evidence supports a dual model of Neolithic spreading into Europe. Philos Trans R Soc Lond B Biol Sci 2742: 161–2167. 10.1098/rspb.2007.0465 PMC2706191 17609193

[47] Talamo S, Richards M (2011) A comparison of bone pretreatment methods for AMS dating of samples >30,000 BP. Radiocarbon 53: 443–449.

[48] Bronk Ramsey C (2009) Bayesian analysis of radiocarbon dates. Radiocarbon 51: 337–360.

[49] Reimer PJ, Baillie MGL, Bard E, Bayliss A, Beck JW, et al (2009) INTCAL09 and MARINE09 radiocarbon age calibration curves, 0–50,000 years cal BP. Radiocarbon 51: 1111–1150.

[50] Brown TA, Nelson DE, Southon JR (1988) Improved collagen extraction by modified Longin method. Radiocarbon 30: 171–177.

[51] DeNiro MJ (1985) Postmortem preservation and alteration of *in vivo* bone collagen isotope ratios in relation to palaeodietary reconstruction. Nature 317: 806–809.

[52] van Klinken GJ (1999) Bone collagen quality indicators for paleodietary and radiocarbon measurement. J Archaeol Sci 26: 687–695.

[53] Andrews RM, Kubacka I, Chinnery PF, Lightowlers RN, Turnbull DM, et al (1999) Reanalysis and revision of the Cambridge reference sequence for human mitochondrial DNA. Nat Genet 23: 147. 10508508 10.1038/13779

[54] Macaulay V, Richards M, Hickey E, Vega E, Cruciani F, et al (1999) The emerging tree of west Eurasian mtDNAs: a synthesis of control-region sequences and RFLPs. Am J Hum Genet 64: 232–249. 9915963 10.1086/302204 PMC1377722

[55] Richards M, Macaulay V, Hickey E, Vega E, Sykes B, et al (2000) Tracing European founder lineages in the Near Eastern mtDNA pool. Am J Hum Genet 67: 1251–1276. 11032788 PMC1288566

[56] Tambets K, Kivisild T, Metspalu E, Parik J, Kaldma K, et al. (2000) The topology of the maternal lineages of the Anatolian and Trans-Caucasus populations and the peopling of Europe: some preliminary considerations. In: Renfrew C, Boyle K, eds. Archaeogenetics: DNA and the population prehistory of Europe. McDonald Institute for Archaeological Research Monograph Series. Cambridge: Cambridge University Press. 219–235.

[57] Garcia-Guixé E, Subirà ME, Marlasca R, Richards MP (2010) δ^13^C and δ^15^N in ancient and recent fish bones from the Mediterranean Sea. J Nordic Arch Sci 17: 83–92.

[58] Richards MP, Jacobi R, Cook J, Pettitt PB, Stringer CB (2005) Isotope evidence for the intensive use of marine foods by Late Upper Palaeolithic humans. J Hum Evol 49: 390–394. 15975629 10.1016/j.jhevol.2005.05.002

[59] Hedges REM (2003) Isotopes and red herrings: comments on Milner et al. and Lidén et al. Antiquity 78: 34–37.

[60] Vika E, Theodoropoulou T (2012) Re-investigating fish consumption in Greek antiquity: results from δ^13^C and δ^15^N analysis from fish bone collagen. J Archaeol Sci 39: 1618–1627.

[61] Deudero S, Pinnegar JK, Polunin NVC, Morey G, Morales-Nin B (2004) Spatial variation and ontogenic shifts in the isotopic composition of Mediterranean littoral fishes. Mar Biol 145: 971–981.

[62] Keenleyside A, Schwarcz H, Stirling L, Lazreg NB (2009) Stable isotopic evidence for diet in a Roman and Late Roman population from Leptiminus, Tunisia. J Archaeol Sci 36: 51–63.

[63] Bocherens H, Drucker D (2003) Trophic level isotopic enrichment of carbon and nitrogen in bone collagen: case studies from recent and ancient terrestrial ecosystems. Int Journal Osteoarchaeol 13: 46–53.

[64] Caramelli D, Lalueza-Fox C, Vernesi C, Lari M, Casoli A, et al (2003) Evidence for a genetic discontinuity between Neandertals and 24,000-year-old anatomically modern Europeans. Proc Natl Acad Sci U.S.A. 100: 6593–6597. 10.1073/pnas.1130343100 PMC164492 12743370

[65] Palma di Cesnola A (2006) L’Aurignacien et le Gravettien ancient de la grotte Paglicci au Mont Gargano. Anthropologie. 110: 355–370.

[66] Mannino MA, Thomas KD (2007) New radiocarbon dates for hunter-gatherers and early farmers in Sicily. Accordia Research Papers 10: 13–34.

[67] D’Amore G, Di Marco S, Di Salvo R, Messina A, Sineo L (2010) Early human peopling of Sicily: evidence from the Mesolithic skeletal remains from Grotta d’Oriente. Ann Hum Biol 37: 403–426. 20412025 10.3109/03014461003712947

[68] Bonfiglio L, Mangano G, Marra AC, Masini F, Pavia M, et al (2002) Pleistocene Calabrian and Sicilian bioprovinces. Geobios (Mem Spec) 24: 30–39.

[69] D’Amore G, Di Marco S, Tartarelli G, Bigazzi R, Sineo L (2009) Late Pleistocene human evolution in Sicily: comparative morphometric analysis of Grotta di San Teodoro craniofacial remains. J Hum Evol 56: 537–550. 19446307 10.1016/j.jhevol.2009.02.002

[70] Masini F, Petruso D, Bonfiglio L, Mangano G (2008) Origination and extinction patterns of mammals in three central Western Mediterranean islands from the Late Miocene to Quaternary. Quat Int 182: 63–79.

[71] Chilardi S, Frayer DW, Gioia P, Macchiarelli R, Mussi M (1996) Fontana Nuova di Ragusa (Sicily, Italy): southernmost Aurignacian site in Europe. Antiquity 70: 553–563.

[72] Gazzoni V, Fontana F (2011) Quelle morte? Quelle vie? Pratiques funéraires et organisation sociale des chasseurs-cueilleurs de la péninsule italienne. Bull Mem Soc Anthropol Paris 23: 52–69.

[73] Mannino MA, Thomas KD (2009) Current research on prehistoric human coastal ecology: Late Pleistocene and Early Holocene hunter-gatherer transitions in north-west Sicily. In: McCartan S, Schulting R, Warren G, Woodman P, eds. Mesolithic Horizons. Oxford: Oxbow Books. 140–145.

[74] Mannino MA, Thomas KD, Tufano E, Tusa S (2012) Lo sfruttamento dei molluschi marini a Grotta di Punta Capperi (Levanzo, Sicilia) tra la fine del Pleistocene e gli inizi dell’Olocene. Atti del 6°Convegno Nazionale di Archeozoologia, Orecchiella 2009: 105–112.

[75] Tinner W, van Leeuwen JFN, Colombaroli D, Vescovi E, van der Knaap WO, et al (2009) Holocene environmental and climatic changes at Gorgo Basso, a coastal lake in southern Sicily, Italy. Quat Sci Rev 28: 1498–1510.

[76] Craig OE, Biazzo M, Tafuri MA (2006) Palaeodietary records of coastal Mediterranean populations. J Mediterr Stud 16: 63–77.

[77] Gallant TW (1985) A fisherman’s tale: an analysis of the potential productivity of fishing in the ancient world. Miscellanea Graeca 7: 1–78.

[78] Binford LR (2001) Constructing frames of reference: an analytical method for archaeological theory building using ethnographic and environmental data sets. Berkeley: University of California Press. 583 p.

[79] Farrugio H, Oliver P, Biagi F (1993) An overview of the history, knowledge, recent and future research trends in Mediterranean fisheries. Scientia Marina 57: 105–119.

[80] Collard M, Buchanan B, Morin J, Costopoulos A (2011) What drives the evolution of hunter-gatherer subsistence technology? A reanalysis of the risk hypothesis with data from the Pacific Northwest. Philos Trans R Soc Lond B Biol Sci 366: 1129–1138. 21357235 10.1098/rstb.2010.0366 PMC3049102

[81] Shennan SJ (2001) Demography and cultural innovation: a model and some implications for the emergence of modern human culture. Camb Archaeol J 11: 5–16.

[82] Siani G, Paterne M, Arnold M, Bard E, Métivier B, et al (2000) Radiocarbon reservoir ages in the Mediterranean Sea and Black Sea. Radiocarbon 42: 271–280.

