# Supplementary material for: Origin and Diet of the Prehistoric Hunter-Gatherers on the Mediterranean Island of Favignana (Ègadi Islands, Sicily)
Source: PLoS One. 2012 Nov 28;7(11):e49802. doi: 10.1371/journal.pone.0049802 (PMC3509116; doi:10.1371/journal.pone.0049802)
Supplement: Table S1 — Clone sequences for the Oriente B sample. (DOC) [file pone.0049802.s001.doc]

**Table S1.Clone sequences for the Oriente B sample.** The first line reports the reference sequence (CRS) with the numbering of the nucleotide positions. Nucleotides identical to the reference sequence are indicated by dots. Clones are identified by an abbreviation and two numbers: the abbreviation refers to the laboratory (F: Florence; B: Barcelona); the first number indicates the extraction; the second number indicates the

PCR.

**1111111111111111111111111111111111111111111111111111111111111111111111111111111111111111111111111111111111111111111111111111111111111111111111111111111111111111111111111111111111111111111111111111111111111111111111111111111111111111111111111111111111111111111111111111111111111111111111111111111111111111111111111111111111111111111111111111111111111111111111111**

**6666666666666666666666666666666666666666666666666666666666666666666666666666666666666666666666666666666666666666666666666666666666666666666666666666666666666666666666666666666666666666666666666666666666666666666666666666666666666666666666666666666666666666666666666666666666666666666666666666666666666666666666666666666666666666666666666666666666666666666666666**

**0000000000000000000000000000000000000000000000000000000000000000000000000000111111111111111111111111111111111111111111111111111111111111111111111111111111111111111111111111111122222222222222222222222222222222222222222222222222222222222222222222222222222222222222222222222222223333333333333333333333333333333333333333333333333333333333333333333333333333333333333**

**2222223333333333444444444455555555556666666666777777777788888888889999999999000000000011111111112222222222333333333344444444445555555555666666666677777777778888888888999999999900000000001111111111222222222233333333334444444444555555555566666666667777777777888888888899999999990000000000111111111122222222223333333333444444444455555555556666666666777777777788888**

**4567890123456789012345678901234567890123456789012345678901234567890123456789012345678901234567890123456789012345678901234567890123456789012345678901234567890123456789012345678901234567890123456789012345678901234567890123456789012345678901234567890123456789012345678901234567890123456789012345678901234567890123456789012345678901234567890123456789012345678901234**

**CRS TTCTTTCATGGGGAAGCAGATTTGGGTACCACCCAAGTATTGACTCACCCATCAACAACCGCTATGTATTTCGTACATTACTGCCAGCCACCATGAATATTGTACGGTACCATAAATACTTGACCACCTGTAGTACATAAAAACCCAATCCACATCAAAACCCCCTCCCCATGCTTACAAGCAAGTACAGCAATCAACCCTCAACTATCACACATCAACTGCAACTCCAAAGCCACCCCTCACCCACTAGGATACCAACAAACCTACCCACCCTTAACAGTACATAGTACATAAAGCCATTTACCGTACATAGCACATTACAGTCAAATCCCTTCTCGTCCCCATGGATGACCCCCCTCAG**

**F_1.1 L15995...........................................T.................................................................H16132**

**F_1.2 ...........................................T.................................................................**

**F_1.3 ...........................................T.................................................................**

**F_2.1 ...........................................T.................................................................**

**F_2.2 ...........................................T............................................................T....**

**B_1.1 L16055........... T...........................................................................H16142**

**B_1.2 ............T...........................................................................**

**B_1.3 ............T.............................G.............................................**

**B_2.1 ............T...........................................................................**

**B_2.2 ............T...........................................................................**

**F_1.1 L16107...........................................................................................................................................................H16261**

**F_1.2 .....................................................................................T.....................................................................**

**F_1.3 ...........................................................................................................................................................**

**F_2.1 ...........................................................................................................................................................**

**F_2.2 ...........................................................................................................................................................**

**F_1.1 L16247...........................................................................................................................................H16402**

**F_1.2 ..................................................C........................................................................................**

**F_1.3 ...........................................................................................................................................**

**F_2.1 ...........................................................................................................................................**

**F_2.2 ...........................................................................................................................................**
